# The Akt-inhibitor Erufosine induces apoptotic cell death in prostate cancer cells and increases the short term effects of ionizing radiation

**DOI:** 10.1186/1748-717X-5-108

**Published:** 2010-11-16

**Authors:** Justine Rudner, Carola-Ellen Ruiner, René Handrick, Hans-Jörg Eibl, Claus Belka, Verena Jendrossek

**Affiliations:** 1 Department of Radiation Oncology, University of Tübingen, Hoppe-Seyler-Straße 3, D-72076 Tübingen, Germany; 2 Department of Molecular Cell Biology, Institute for Cell Biology, University of Duisburg-Essen, D-45122 Essen, Germany; 3 Institute of Pharmaceutical Biotechnology, University of Applied Sciences, Hubertus-Liebrecht-Str. 35, 88400 Biberach, Germany; 4 Max-Planck-Institute for Biophysical Chemistry, Am Fassberg 11, 37077 Göttingen, Germany; 5 Clinic of Radiation Oncology, Marchioninistraße 15, D-81377 München, Germany

## Abstract

**Background and Purpose:**

The phosphatidylinositol-3-kinase (PI3K)/Akt pathway is frequently deregulated in prostate cancer and associated with neoplastic transformation, malignant progression, and enhanced resistance to classical chemotherapy and radiotherapy. Thus, it is a promising target for therapeutic intervention. In the present study, the cytotoxic action of the Akt inhibitor Erufosine (ErPC3) was analyzed in prostate cancer cells and compared to the cytotoxicity of the PI3K inhibitor LY294002. Moreover, the efficacy of combined treatment with Akt inhibitors and ionizing radiation in prostate cancer cells was examined.

**Materials and methods:**

Prostate cancer cell lines PC3, DU145, and LNCaP were treated with ErPC3 (1-100 µM), LY294002 (25-100 µM), irradiated (0-10 Gy), or subjected to combined treatments. Cell viability was determined by the WST-1 assay. Apoptosis induction was analyzed by flow cytometry after staining with propidium iodide in a hypotonic citrate buffer, and by Western blotting using antibodies against caspase-3 and its substrate PARP. Akt activity and regulation of the expression of Bcl-2 family members and key downstream effectors involved in apoptosis regulation were examined by Western blot analysis.

**Results:**

The Akt inhibitor ErPC3 exerted anti-neoplastic effects in prostate cancer cells, however with different potency. The anti-neoplastic action of ErPC3 was associated with reduced phosphoserine 473-Akt levels and induction of apoptosis. PC3 and LNCaP prostate cancer cells were also sensitive to treatment with the PI3K inhibitor LY294002. However, the ErPC3-sensitive PC3-cells were less susceptible to LY294002 than the ErPC3-refractory LNCaP cells. Although both cell lines were largely resistant to radiation-induced apoptosis, both cell lines showed higher levels of apoptotic cell death when ErPC3 was combined with radiotherapy.

**Conclusions:**

Our data suggest that constitutive Akt activation and survival are controlled by different different molecular mechanisms in the two prostate cancer cell lines - one which is sensitive to the Akt-inhibitor ErPC3 and one which is more sensitive to the PI3K-inhibitor LY294002. Our findings underline the importance for the definition of predictive biomarkers that allow the selection patients that may benefit from the treatment with a specific signal transduction modifier.

## Introduction

Prostate cancer is the most commonly diagnosed malignancy in men. Radical prostatectomy, hormone ablation therapy, and radiotherapy are available for treatment of localized stages yielding >50% of local control [1,2]. Radiotherapy is also an integral part of treatment protocols for inoperable locally advanced prostate cancer. Despite the use of classical chemotherapy (mainly taxanes), hormone ablation therapy, radiopharmaceuticals, and refined radiation methods, no curative treatment for advanced stages is available to date. Thus, novel approaches are needed particularly for the treatment of patients with hormone-refractory disease [3,4].

Malignant progression is mostly associated with resistance to cell death induction by chemo- and radiotherapy. Therefore, molecular targeting agents that overcome cell death resistance or increase the sensitivity of malignant cells to the cytotoxic action of chemo- or radiotherapy may be suited to improve treatment outcome in localized disease and advanced stages. Altered signaling pathways within the tumor cells that affect tumor cell survival are in focus for the development of innovative anticancer drugs. The PI3K/Akt pathway is one of the most important survival signaling cascades altered in human solid tumors including prostate cancer [5,6]. In normal cells, this pathway transmits growth and survival signals from cell surface receptors to promote cell survival in response to cellular stress. An aberrant activation of growth factor receptors, activating mutations of PI3K, or the inactivation of the tumor suppressor phosphatase and tensin homolog on chromosome ten (PTEN) which counteracts PI3K lead to an constitutive activation of the PI3K/Akt pathway. Up-regulated activity of the kinase Akt is associated with malignant transformation characterized by accelerated tumor growth, metastasis, and angiogenesis. Moreover, activated Akt decreases sensitivity of tumor cells to chemotherapy and radiotherapy by increasing the threshold for cell death induction [7]. Therefore, the survival kinase Akt attracted major attention for the development of molecularly targeted approaches for the treatment of human solid tumors including prostate cancer and overcoming resistance to standard genotoxic chemo- and radiotherapy. Importantly, Akt is embedded into a highly complex network of upstream regulators and downstream effector proteins and it is still unclear whether targeting the kinase itself or its regulators/modulators will provide the most pronounced anti-neoplastic effect.

In our previous investigations, we could confirm that malignant tissues from patients with localized prostate cancer are frequently characterized by increased expression of phospho-Akt (Ser473). Interestingly, only in a subgroup of the patients increased expression of phospho-Akt correlated with loss or inactivation of its upstream regulator PTEN [8]. Moreover, we found a substantial heterogeneity in the expression and phosphorylation levels of the Akt-downstream targets forkhead transcription factor like 1 (FKHRL1), glycogen synthase kinase-3β (GSK3β), and mammalian target of rapamycin (mTOR). Thus, the existence of different molecular subgroups with distinct sensitivity to small molecule inhibitors of the PI3K/Akt-pathway and radiotherapy can be assumed [8].

Alkylphosphocholines are lysophospholipid-like inhibitors of the signal transduction pathways with anti-neoplastic properties. In contrast to classic genotoxic chemotherapy and radiotherapy, these lipophilic drugs target cellular membranes and interfere with membrane lipid composition and the formation of lipid second messengers, thereby affecting the growth, cell cycle progression, and survival of tumor cells without any direct effects on the genome [9]. The use of two clinically relevant derivatives, the oral drug perifosine and the prototypic intravenously applicable ErPC3, in preclinical and clinical investigations is based on their ability to induce apoptosis in tumor cells and their ability to increase cytotoxic efficacy of chemotherapy and radiotherapy in preclinical investigations [10-12]. Induction of apoptosis by ErPC3 and related drugs occurs mainly via the mitochondrial pathway which is controlled by several pro- and anti-apoptotic members of the Bcl-2 protein family [13,14]. However, particularly in leukemic cells, the extrinsic pathway can also be involved [15]. The cytotoxic action of synthetic phospholipid analogs relies on their ability to affect specific signaling processes in the tumor cells such as the proapoptotic stress-activated protein kinase (SAPK)/c-jun-NH_2_-terminal kinase (JNK) pathway, the prosurvival PI3K/Akt pathway, and the mitogen-activated protein kinase (MAPK)/extracellular signal-regulated kinase (ERK) pathway [9].

Here we evaluated of the anti-neoplastic activity of the putative Akt inhibitor ErPC3 in different prostate cancer cell lines *in vitro*. ErPC3´s anti-neoplastic action was compared to that of the known PI3K-inhibitor LY294002. In addition, we compared the anti-neoplastic effects of ErPC3 and LY294002 in combination with ionizing radiation.

## Materials and methods

### Chemicals and drugs

ErPC3 was synthesized by H. Eibl, Max Planck Institute of Biophysical Chemistry, (Goettingen, Germany) and dissolved in RPMI 1640 medium at 10 mg/ml. LY294002 was obtained from Cell Signaling (Frankfurt, Germany). Rabbit antibodies against PARP, caspase-3, Akt, phospho-Akt (Ser473), Bax, Mcl-1, and Bcl-xL were purchased from Cell Signaling (Frankfurt, Germany), the rabbit anti-Bak NT antibody was from Upstate (Biomol, Hamburg, Germany). Mouse anti-ß-Actin was obtained from Sigma-Aldrich (Deisenhofen, Germany). HRP-conjugated anti-rabbit and anti-mouse secondary antibodies were from Amersham-Biosciences (Freiburg, Germany). All other chemicals were purchased from Sigma-Aldrich (Deisenhofen, Germany) if not otherwise specified.

### Cell lines and cell culture

The prostate cancer cell lines LNCaP (p53 wild type, androgen-dependent, highly differentiated), PC3 (p53-/-, androgen-independent, poorly differentiated), and DU145 (p53 mutant, androgen-independent, moderately differentiated) were obtained from ATCC (Bethesda, Maryland, USA). For all experiments cells were grown in RPMI 1640 medium supplemented with 10% (v/v) fetal calf serum (Gibco Life Technologies, Eggenstein, Germany) and maintained in a humidified incubator at 37°C and 5% CO_2_.

### Treatment of cells

Cells were irradiated at room temperature with 6 MV photons from a linear accelerator (LINAC SL25 Phillips) at a dose rate of 4 Gy/min at room temperature. A single dose of 2 Gy, 5 Gy, or 10 Gy was applied. ErPC3 was used at a final concentration of 1-100 µM, the PI3K inhibitor LY294002 was used at a final concentration of 25-100 µM.

### Cell proliferation and viability assay

10^3^, 2 × 10^3 ^or 3 × 10^3 ^cells/well were seeded in 96 well plates and left to attach at 37°C over night. Subsequently, cells were stimulated as described above. Cell survival was measured at indicated time points by adding 10 µl of a 1:3 (v/v) diluted ready to use WST-1 cell proliferation reagent stock solution (Roche, Mannheim). Samples were incubated for 60-240 min and absorption was measured with ANTHOS^® ^MTP reader (Anthos Mikrosystheme GmbH, Krefeld, Germany) at 450 nm wavelength using a 620 nm reference filter. After subtraction of the background absorption, the mean values of the untreated control cells were set as 100%.

### DNA fragmentation

Nuclear fragmentation was determined after staining the cells with 5 µg/mL propidium iodide in a hypotonic buffer containing 0.1% sodium citrate and 0.1% Triton X-100 for 1 h at room temperature. The stained cells were detected in channel 2 employing a FACS Calibur flow cytometer and the Cell Quest software (Becton Dickinson, Heidelberg, Germany). Flow cytometric analysis was performed using FCS Express software (De Novo Software, Los Angeles, CA, USA).

### Western blot

Cells were lysed in lysis buffer containing 50 mM HEPES pH7.5, 150 mM NaCl 1% Triton X-100, 1 mM EDTA, 10 mM sodium pyrophosphate, 10 mM NaF, 2 mM Na_3_VO_4_, 100 mM PMSF, 5 µg/ml Aprotinin, 5 µg/ml Leupeptin, and 3 µg/ml Pepstatin. After removing insoluble material by centrifugation for 10 min at 13 000 r.p.m., the protein concentration was estimated in the supernatant using the Bio-Rad protein assay (Bio-Rad, Munich, Germany) according to the manufacturer's protocol. Lysates were separated by SDS-PAGE under reducing conditions before transfer onto PVDF-membranes (Roth, Karlsruhe, Germany). Equal protein loading was confirmed by Ponceau S staining. Blots were blocked in TBS buffer containing 0.05% Tween 20 and 5% non-fat dried milk for 1 h at room temperature. The membrane was incubated over night at 4°C with the respective primary antibodies. After repeated washings with TBS/Tween-20 (0.05%) the membranes were incubated with the secondary antibody for 1 h at room temperature before repeating the washing with TBS/Tween-20 (0,05%). Detection of antibody binding was performed by enhanced chemoluminescence according to the manufacturer's protocol (ECL Western blotting analysis system, GE Healthcare/Amersham-Biosciences, Freiburg, Germany).

### Data analysis

Experiments were at least performed in triplicate. Data were represented as means ± SD (DNA fragmentation and cell proliferation/viability assay) or as one representative out of three similar experiments (Western Blot). Statistical significance was calculated by ANOVA test using GraphPad Software (San Diego, CA, USA, http://www.graphpad.com).

## Results

### Antineoplastic efficacy of ionizing radiation and ErPC3 in prostate cancer cell lines

**Figure 1:**
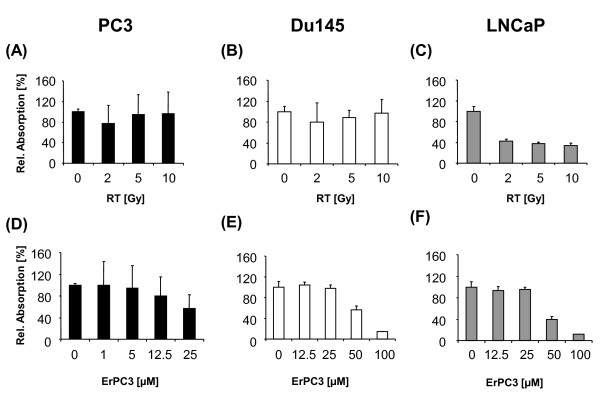
**Anti-neoplastic effects of ErPC3 and ionizing radiation on prostate cancer cells**. The prostate cancer cell lines PC3, DU145, and LNCaP, were irradiated (RT) with 2, 5, or 10 Gy (A-C) or treated with 1-100 µM ErPC3, as indicated (D-F). 48 h after treatment a WST-1 Assay was performed. The absorption correlates with the number of viable cells and was normalized to that of untreated controls. PC3 (A) and DU145 (B) were not affected by ionising radiation whereas the number of viable LNCaP cells was reduced 48 h after irradiation (C). All cell lines responded to ErPC3-treatment in a concentration-dependent manner. The androgen-independent cell line PC3 was most sensitive to ErPC3 (D). 25 µM ErPC3 reduce the number of viable PC3 cells by approximately 50% whereas 50 µM ErPC3 were needed to affect the viability of DU145 (E) and PC3 cells (F).

In a first step, the anti-neoplastic effects of ErPC3 and ionizing radiation alone were analyzed in three different prostate cell lines. For this, PC3, DU145, and LNCaP cells were subjected to single doses of ionizing radiation between 2 Gy and 10 Gy or treated with different concentrations of ErPC3 (1 µM to 100 µM). 48 h later, cells were subjected to the WST-1 proliferation/viability assay. In LNCaP cells, ionizing radiation reduced the number of viable cells already at low doses (Figure 1C). In contrast, PC3 and DU145 cells remained almost unaffected by radiation treatment, even when higher radiation doses (5 or 10 Gy) were applied (Figure 1A and Figure 1B). Interestingly, PC3 cells were highly sensitive to treatment with ErPC3: we observed a 50% reduction in the number of viable cells already upon treatment with 25 µM ErPC3 (Figure 1D). However, the same drug concentration failed to reduce the number of viable DU145 and LNCaP cells (Figure 1E and Figure 1F). Both cell types were only affected by treatment with ErPC3 when concentrations of 50 µM ErPC3 or higher were used.

### Apoptosis-induction by ErPC3 and ionizing radiation in prostate cancer cell lines

**Figure 2:**
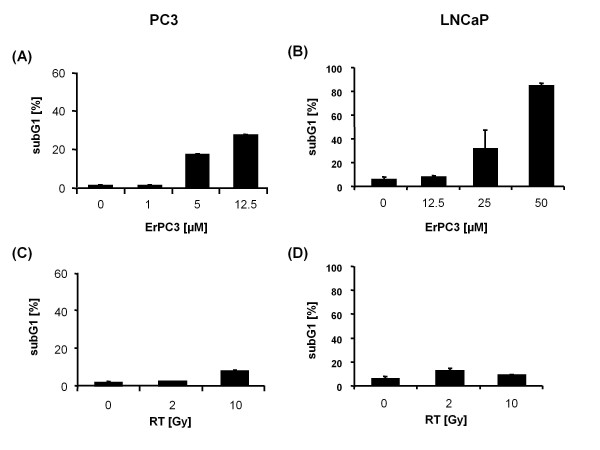
**Apoptosis induction in response to ErPC3 and ionizing radiation**. PC3 and LNCaP cells were treated with 1-50 µM ErPC3 or irradiated with a single dose of 2 or 10 Gy. 48 h later, cells were stained with propidium iodide in a hypotonic citrate buffer containing Triton X-100 and subjected to flow cytometric analysis to estimate DNA fragmentation which occurs upon induction of apoptosis. 5 µM ErPC3 were sufficient to induce DNA fragmentation in PC3 cells (A), whereas 25 µM ErPC3 were required to trigger apoptotic DNA-fragmentation in LNCaP cells (B). Ionizing radiation up to 10 Gy did not induce DNA-fragmentation above a background level in PC3 (C) and LNCaP cells (D).

**Figure 3:**
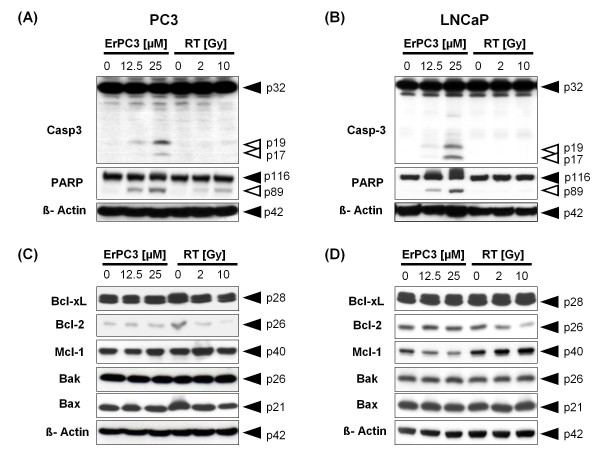
**Activation of caspase-3 and regulation of Bcl-2 protein family members in response to ErPC3-treatment and irradiation**. PC3 and LNCaP cells were treated with 0-25 µM ErPC3 or irradiated with 2 or 10 Gy. Cells lysates were generated 48 h after treatment, separated by electrophoresis, and protein expression was subsequently analyzed by western blotting. Both cells lines showed a concentration-dependent activation of caspase-3 in response to ErPC3-treatment (A, B). In PC3 cells, cleavage of the caspase-3 substrate PARP could already be detected after treatment with 12.5 µM ErPC3; PARP-cleavage was accompanied by a weak activation of caspase-3 detectable upon treatment with 12.5 µM ErPC3 (A). A weak cleavage of caspase-3 and PARP was also observed when LNCaP cells were treated with 12.5 µM ErPC3, but cleavage was clearly visible after treatment with 25 µM ErPC3 (B). No caspase-3 activation and PARP cleavage was observed in response to ionizing radiation. No change of protein levels of the pro-apoptotic Bak and Bax and the anti-apoptotic Bcl-xL was observed upon irradiation or in response to treatment with ErPC3 (C, D). A slight reduction in the levels of antiapoptotic Bcl-2 was observed upon irradiation in LNCaP and PC3 cells, whereas treatment with ErPC3 reduced the levels of the anti-apoptotic Mcl-1 in LNCaP cells. However, the changes of Mcl-1 expression levels did not correlate with the sensitivity of LNCaP cells to ErPC3.

The WST-1 assay mirrors just the number of viable cells at a specific time point, but does not indicate whether the therapy effects observed are due to inhibition of proliferation, cell death induction, or both. Therefore, in a next step, we examined whether the anti-neoplastic effects of ErPC3 and ionizing radiation include induction of cell death, in particular apoptosis. These investigations were performed in the highly ErPC3-sensitive PC3 cells and the less ErPC3-sensitive LNCaP cells using flow cytometric detection of apoptosis-related nuclear fragmentation (Figure 2). As shown in Figure 2A, ErPC3 induced prominent DNA fragmentation in PC3 cells already at low dose treatment (5 µg/mL ErPC3). In contrast, 25 µM ErPC3 were needed to trigger a significant amount of cells with nuclear fragmentation in LNCaP cells (Figure 2B). So far, these observations were in line with the data obtained from the WST-1 viability assay. As expected from the results of the WST-1 assay, we hardly detected any apoptosis in PC3 cells in response to ionizing radiation (Figure 2C). However, despite reducing the number of viable cells in the WST-1 assay, ionizing radiation did not induce significant apoptotic nuclear fragmentation in LNCaP cells (Figure 2D). In line with these findings, caspase-3 activation - as indicated by p19 and p17 cleavage products - and cleavage of the caspase-3 substrate Poly-(ADP-ribose)-Polymerase (PARP) was only observed in the lysates of ErPC3-treated prostate cancer cells but not in the lysates of irradiated prostate cancer cells (Figure 3A and Figure 3B). These results indicated that ErPC3 is able to trigger apoptosis in PC3 and LNCaP prostate cancer cell lines, although with different potency. In contrast, the anti-neoplastic effects of ionizing radiation in LNCaP cells did not involve apoptosis induction implicating a role of proliferation inhibition or the induction of non-apoptotic or delayed cell death modes.

### Impact of ErPC3 and ionizing radiation on the levels of Bcl-2 proteins

As shown in previous investigations, ErPC3 induces apoptosis via the intrinsic mitochondrial pathway [16]. We therefore next examined whether the differences in apoptosis sensitivity of LNCaP and PC3 cells may be related to differences in the basal levels or treatment-induced changes in the expression of several proteins of the Bcl-2 family known to function as key regulators of the mitochondrial homeostasis and intrinsic apoptosis. As shown in Figs. 3C and 3D, PC3 and LNCaP cells expressed pro-apoptotic Bax and Bak, but the expression levels of those pro-apoptotic effector proteins were not affected by treatment with ErPC3 or ionizing radiation. LNCaP and PC3 cells expressed the anti-apoptotic Bcl-2 proteins Bcl-xL, Mcl-1, and Bcl-2, although at different levels: Both cell lines expressed a high amount of Bcl-xL, and an intermediate amount of Mcl-1, whereas expression levels of Bcl-2 were intermediate (LNCaP-cells) or low (PC3-cells) (Figure 3C and 3D). Treatment with ErPC3 did not affect the protein levels of Bcl-xL and Bcl-2 in LNCaP and PC3 cells, whereas ionizing radiation triggered a decrease in the levels of Bcl-2 in both cell lines. Moreover, ErPC3-treatment decreased the levels of Mcl-1 in LNCaP cells. Thus, in LNCaP cells the down-regulation of the two anti-apoptotic Bcl-2 proteins may contribute to the antineoplastic effects of ErPC3 and radiotherapy. In contrast, the radiation-induced down-modulation of the very low Bcl-2-levels may be of minor importance for the regulation of cell survival in PC3 cells. The differential effect on Mcl-1 expression does not provide a molecular basis for the distinct sensitivities of PC3 and LNCaP cells to ErPC3-treatment since the levels of Mcl-1 remained unaffected in the highly ErPC3-sensitive PC3 cells.

### Impact of ErPC3 on the phosphorylation state of protein kinase B (Akt)

**Figure 4:**
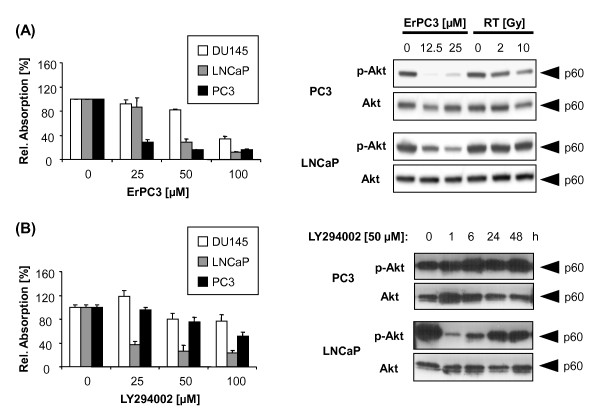
**Differential effects of ErPC3 and LY294002 on prostate cancer cell survival and p-Akt levels**. (A, B, left panels) DU145, LNCaP, and PC3 cells were treated with solvent controls or 25-100 µM ErPC3 or 25-100 µM LY294002. 48 h later a WST-1 assay was performed to quantify the number of viable cells. PC3 cells were most sensitive to treatment with ErPC3 (A, left panel), whereas LNCaP cell were most susceptible to LY294002-treatment (B, left panel). Western blot analysis of lysates generated from PC3 cells 48 h after treatment with 0-25 µM ErPC3 showed a massive reduction of Akt-phosphorylation at serine 473 (p-Akt) whereas almost no reduction of p-Akt was found 48 h after irradiation with 2 or 10 Gy (A, right panel). ErPC3 also reduced p-Akt levels in LNCaP cells however with lower potency (A, right panel). Western blot analysis of lysates generated 0-48 h after treatment with 50 µM LY294002 showed a massive down-modulation of p-Akt-levels in LNCaP cells within 1 h after treatment; still, a considerable reduction in p-Akt levels could be detected 48 h after treatment. In contrast, LY294002 failed to reduce p-Akt levels in PC3 cells at any time point measured (B, right panel).

The apoptosis threshold of tumor cells is controlled by various survival pathways including the PI3K/Akt pathway. This pathway is frequently deregulated in prostate cancer patients. It has been shown earlier that the anti-neoplastic action of ErPC3 and related compounds is associated with the inhibition of Akt [14,17,18]. We therefore next evaluated the potential of ErPC3 to inhibit the survival kinase Akt in PC3 and LNCaP prostate cancer cells. Moreover, we compared the effects of ErPC3 to the effects of the PI3K inhibitor LY294002. LY294002 inhibits the upstream kinase PI3K thereby preventing the activation of Akt. PC3, LNCaP, and DU145 cells were treated with 25-100 µM ErPC3 or LY294002 for 48 h before analyzing the number of viable cells by the WST-1 assay (Figure 4). As already depicted in Figure 1A, PC3 cells were most sensitive to the treatment with ErPC3. In these cells treatment with 25 µM ErPC3 was sufficient to reduce the number of viable PC3 cells by more than 50%, whereas 50µM and 100 µM ErPC3 were required to obtain a similar response in LNCaP and DU145 cells, respectively (Figure 4A, left panel). The observed differences of the relative absorption in this experiment as compared to that in Figure 1 are due to slightly different experimental procedures. Higher cell numbers and longer incubation time with WST-1 resulted in an increased absolute absorption and smaller error bars. A completely different picture was obtained when testing the anti-neoplastic potency of the PI3K inhibitor LY294002 (Figure 4B, left panel). In these investigations, LNCaP cells turned out to be the most sensitive of the three prostate cancer cell lines. 25 µM LY294002 reduced the number of viable LNCaP cells by more than 50% whereas 100 µM LY294002 were required to exhibit a similar inhibitory potential in PC3 cells. Again, DU145 cells displayed only very modest sensitivity to the inhibition of the PI3K/Akt pathway (Figure 4B, left panel).

The differences in the sensitivity may be due to a distinct potential of the drugs to interfere with Akt signaling. We therefore next examined treatment-induced changes in the levels of phospho-serine 473 Akt (p-Akt). Phosphorylation at serine 473 is required to obtain full activation Akt. As shown in Figure 4A (right panel), treatment with ErPC3 caused a dramatic reduction in the levels of p-Akt in PC3 cells. A less pronounced but still remarkable reduction in p-Akt was observed in LNCaP correlating with the different sensitivity of the two cell lines to ErPC3. The PI3K inhibitor LY294002 (50µM) largely reduced p-Akt-levels in LNCaP cells. Maximal inhibition was already observed 1 h after addition of LY294002 to LNCaP cells, but p-Akt was still reduced 2 days later (Figure 4B right panel). Interestingly, in PC3 cells treatment with LY294002 was without effect on the phosphorylation state of Akt. Even 48 h after treatment, p-Akt levels remained unaffected (Figure 4B right panel). Because PC3 cells were highly resistant to the treatment with LY294002, these observations suggest that a down-regulation of p-Akt may be required for the anti-neoplastic action of small molecule inhibitors of the PI3K/Akt pathway in prostate cancer cells.

### Combined effects of ErPC3 and ionizing radiation in prostate cancer cell lines

**Figure 5:**
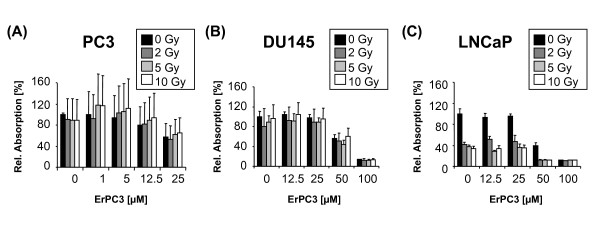
**Anti-neoplastic effect of combined treatment with ErPC3 and ionizing radiation**. PC3 cells (A), DU145 cells (B), and LNCaP cells (C) were treated with increasing concentrations of ErPC3 (1-100 µM) and ionizing radiation (2, 5, 10 Gy) alone or in combination as indicated. Cell viability was analyzed 48 h after treatment by using the WST-1 assay. PC3 and DU145 cell did not respond to irradiation alone but responded to single treatment with ErPC3 (A, B). The anti-neoplastic effects of the combination were mainly attributed to the effects of ErPC3 (A, B). In contrast, LNCaP cells were highly sensitive to treatment with radiation alone, as well as to ≥ 50 µM ErPC3 (C). When LNCaP cells were treated with subtoxic ErPC3-concentrations in combination with irradiation, the reduction in the number of viable cells was mainly due to ionizing radiation (C). However, the cell viability was further reduced when LNCaP cells were treated with toxic ErPC3-concentrations (≥ 50µM) in combination with irradiation.

**Figure 6:**
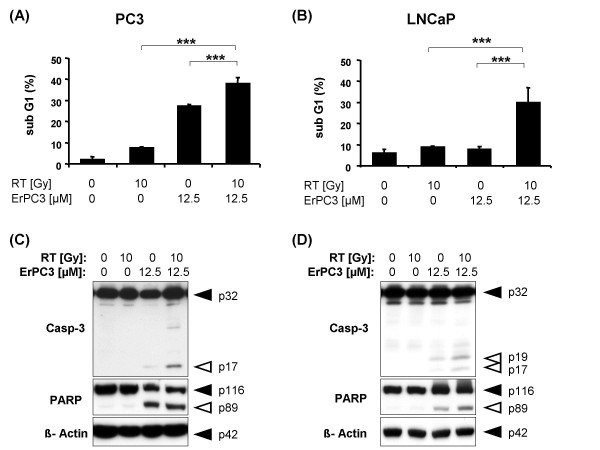
**Combined effects of ErPC3 and ionizing radiation on apoptosis induction in prostate cancer cell lines**. PC3 and LNCaP cells were irradiated with 10 Gy, treated with 12.5 µM ErPC3 or both treatments were combined. DNA fragmentation (A, B) and caspase activation (C, D) were analyzed 48 h later. (A) Approximately 30% of PC3 cells showed DNA fragmentation after a single treatment with ErPC3, whereas radiation-induced apoptosis was below 10%. The amount of apoptotic cells significantly increased when cells were subjected to combined treatment (data show means ± SD; n = 3; ***: p < 0,001). (B) Although a single therapy with ionizing radiation or ErPC3 did not induce apoptotic DNA fragmentation in LNCaP cells, the combination of both treatments resulted in apoptosis levels comparable to that in PC3 cells. The results were confirmed by Western blotting analyzing caspase-3 and PARP cleavage (C, D). Cleavage of caspase-3 (PC3 and LNCaP cells) and PARP (LNCaP cells) was more effective when ErPC3 and ionizing radiation were combined (C, D).

Up to now our data revealed that ErPC3 is a potent inhibitor of Akt even in cells that are highly refractory to inhibitors acting upstream of Akt in the same pathway. Because inhibition of Akt can lower the threshold for cell death induction, we next examined whether an inhibition of the Akt survival pathway by ErPC3 sensitizes the cells to the cytotoxic effects of ionizing radiation. Cells were exposed to different ErPC3 concentrations in combination with 0, 2, 5, or 10 Gy. 48 h later the number of viable cells was determined using the WST-1 assay (Figure 5). While treatment with ionizing radiation was without effect, treatment with ErPC3 resulted in a concentration-dependent decrease in the number of viable PC3 and DU145 cells. Additional irradiation of the cells did not significantly enhance the anti-neoplastic effects compared to single treatment with ErPC3 (Figure 5A and Figure 5B). In LNCaP cells, irradiation with 2 to 10 Gy or treatment with 50 to 100 µM ErPC3 led to a prominent reduction in the number of viable LNCaP cells. When irradiation was combined with subtoxic concentrations of ErPC3, the anti-neoplastic effects of the combined treatment were mainly due to the effects of ionizing radiation (Figure 5C). Only when using a toxic concentration of ErPC3 (50µM), the combination of drug treatment and ionizing radiation was able to further increase the anti-neoplastic effects compared to single treatment with ErPC3 or irradiation alone. As already mentioned above, the Wst-1 test is suited to determine the number of viable cells but does not provide information about the contribution of cytostatic or cytotoxic effects of the treatment under investigation. Therefore, to gain insight into a combination effect on apoptosis induction we subsequently assessed DNA-fragmentation by using flow cytometry and caspase-activation by using Western blot analysis. In PC3 cells treatment with 12.5 µM ErPC3 alone effectively induced apoptosis whereas irradiation alone was almost without effect. The combination of 12.5 µM ErPC3 and 10 Gy led to a small but significant increase in the apoptosis rate compared to either treatment alone (Figure 6A). In LNCaP cells, combined treatment with 12.5 µM ErPC3 and ionizing radiation (10 Gy) induced significant apoptosis although, when applied alone, neither irradiation nor ErPC3 induced apoptotic DNA-fragmentation (Figure 6B). The increased pro-apoptotic effects of ionizing radiation in combination with ErPC3 were also detected when analyzing apoptosis signaling by Western blotting: In both cell types, activation of caspase-3 was increased upon combined treatment compared to either treatment alone (Figure 6C and 6D).

Taken together, our results show that the Akt-inhibitor ErPC3 increases radiation-induced apoptosis in prostate cancer cells. The most prominent combination effects were obtained in LNCaP cells that did not show any apoptosis in response to treatment with irradiation alone.

## Discussion

Although improved screening methods allow a diagnosis of prostate cancer at an early stage, it still remains one major cause of death in men in industrialized countries. In particular, no curative treatment is available to date upon progression to androgen-independent and metastatic disease. Therefore, current research focuses on signal transduction inhibitors to improve the treatment outcome. Based on its suggested role in tumor progression and resistance to standard chemotherapy and radiotherapy, the PI3K/Akt pathway constitutes an attractive therapeutic target in prostate cancer [8,19,20]. Many pharmaceutical companies hunt for novel drugs that interact with the Akt pathway [7]. A group of these, the synthetic phospholipid derivatives perifosine and erucylphosphohomocholine (ErPC3) constitute interesting compounds as they affect intracellular signaling cascades upon primary interaction with cellular membranes [9]. Nude mice treated repeatedly with ErPC3 displayed no major side effects [21]. Here, we show for the first time that the paradigmatic intravenously applicable alkylphosphocholine ErPC3 potently induces apoptosis in prostate cancer cells *in vitro*. These findings corroborate earlier reports on high efficacy of ErPC3 in human glioblastoma, lymphoma, leukemia, and breast cancer cells *in vitro *[10,14,22-25]. Notably, the hormone-independent cell line PC3 was even more sensitive to the cytotoxic effects of ErPC3 than the hormone-responsive cell line LNCaP. In both cell lines, the cytotoxic efficacy of ErPC3 was associated with a reduction in the cellular levels of phospho-Serine 473 Akt (p-Akt) which is indicative for the activation state of this survival kinase. Again, the dephopshorylation of Akt by ErPC3 was more prominent in the highly ErPC3-sensitive PC3 cells compared to the less responsive LNCaP cells. A potent p-Akt-inhibitory action of ErPC3 in association with prominent cytotoxic drug activity was also observed in human malignant glioma cell lines in our earlier investigations [12,14,25]. Similarly, malignant glioma cells are also mostly characterized by an increased activation of the PI3K/Akt survival pathway. Our data also corroborate earlier reports about potent Akt-inhibition by the orally available alkylphosphocholine perifosine in different solid tumor cells *in vitro *including lung and prostate cancer [12,14,17,25,26]. Altogether, these observations suggest a role of Akt-inhibition for the cytotoxic actions of ErPC3 and related compounds when used as single drugs. However, it cannot be excluded that additional effects of ErPC3 and related compounds may contribute to their antineoplastic effects. Here, among others the pro-apoptotic SAPK/JNK pathway, the MAPK/ERK pathway, the sphingolipid pathway, the cell cycle controlling retinoblastoma protein, the F(0)F (1)-ATP synthase, and protein phosphatase 2A have been described as important drug targets [9,27-29].

Interestingly, the anti-neoplastic activity of the PI3K inhibitor LY294002 on the prostate cancer cells differed considerably from the effects of ErPC3: LY294002 exerted its strongest anti-neoplastic effects in LNCaP cells whereas the highly ErPC3-sensitive PC3 cells responded only to high LY294002 concentrations. Importantly, LY294002-treatment reduced the phosphorylation of Akt only in the LY294002-sensitive LNCaP cells but not in PC3 cells with low sensitivity to LY294002. Thus, the antineoplastic activity of ErPC3 and LY294002 in prostate cancer cells correlated with their potency to reduce p-Akt levels. Because ErPC3 and LY294002 act at two distinct levels of the PI3K/Akt-pathway to reduce Akt-activity, the distinct potency of ErPC3 and LY294002 to inhibit Akt-activity in PC3 and LNCaP cells suggests that two distinct but functionally equivalent molecular changes promote up-regulated activity of Akt in LNCaP and PC3 cells. This is reminiscent of our recent observation in tissue probes of patients with localized prostate cancer: In the patients tissues, up-regulated activity of Akt occured as a consequence of PTEN-loss, PTEN-inactivation, or by PTEN-independent mechanisms [8]. These observations may at least partially explain the finding that the ErPC3-related drug perifosine was only active in a subgroup of patients with recurrent androgen-sensitive tumors [30].

In this regard, the PI3K-mediated formation of phospatidylinositol-3,4,5-triphophate (PIP_3_) plays a major role in growth factor mediated activation of Akt. This process is counteracted by the action of the tumor suppressor PTEN which is lost or inactivated in a variety of solid human tumors, including prostate cancer. Increased levels of PIP_3 _trigger the recruitment of phosphatidylinositide-dependent serine/threonine kinase 1 (PDK1) and Akt to the cytoplasmic membrane where PDK1 phosphorylates Akt on threonine 308. An additional phosphorylation on serine 473 is required to fully activate Akt. Phosphorylation on threonine 308 obviously precedes phosphorylation on serine 473 but phosphorylation on serine 473 seems to be independent of PDK1. Although several kinases, such as integrin-linked kinase, DNA-dependent protein kinase (DNA-PK), and the mTOR/Rictor-complex have been proposed to function as so-called "PDK-2" [31-34], the identity of the serine-473 kinase is still controversial [35]. There is accumulated evidence that LY294002 interferes with the activation of Akt by inhibiting its upstream regulator PI3K. In contrast, membrane-targeted alkylphosphocholines like ErPC3 interfere with membrane composition thereby affecting the recruitment of Akt to the plasma membrane which is a prerequisite for its activation by PDK1 [17]. On the basis of this mechanism of action, ErPC3 and related compounds would even be effective in cells where the high activity of Akt is caused by a constitutively active PI3K that is not inhibited by LY294002.

In our hands, treatment with LY294002 resulted in a rapid and consistent downregulation of p-Akt levels in the highly LY294002-sensitive LNCaP cells. ErPC3-treatment also reduced p-Akt levels in LNCaP cells to a substantial amount. The decrease in p-Akt was accompanied by the induction of cell death by both compounds. This suggests that in LNCaP cells the constitutive activation of the survival kinase Akt occurs downstream of an overactive PI3K that is inhibited by both, the PI3K inhibitor LY294002 and the Akt-inhibitor ErPC3. In PC3 cells howerver, only ErPC3 reduced p-Akt and induced cell death to a significant amount when concentrations below 50 µM were used. This suggests that the high p-Akt-levels in PC3 cells rely on a LY294002-insensitive but ErPC3-sensitive mechanism. Thus, PC3 cells may express a mutant PI3K that is insensitive to inhibition by LY294002. Alternatively, Akt-activation in PC3 cells may occur independently from PI3K, e.g. by aberrant activation of Akt-activating kinases or by loss or inactivation of p-Akt phosphatases.

There is accumulated evidence that constitutive activation of the PI3K/Akt pathway interferes with the cytotoxic action of ionizing radiation. On the other hand, it is known from earlier investigations that the antineoplastic efficacy of ErPC3 is increased in human tumor cells when the drug is combined with genotoxic agents like cytarabine, idarubicine or etoposide, or with ionizing radiation, respectively [10,23]. Therefore, in a final set of *in vitro *experiments, we analyzed whether treatment with the Akt-inhibitor ErPC3 would increase the short-time antineoplastic effects of ionizing radiation in the prostate cancer cell lines. Combined treatment with ErPC3 and 2, 5 or 10 Gy reduced the number of viable LNCaP, PC3 and DU145 cells as determined by the WST-1 test.

In PC3 and DU145 cells the antineoplastic effects of the combination treatment could mainly be attributed to the concentration-dependent effects of ErPC3. Although in the WST-1 assay additional irradiation did not cause a further decrease in viable DU145 or PC3 cells, a small but significant increase in the amount of apoptotic PC3 cells could be detected by flow cytometry when ErPC3-treatment was combined with ionizing radiation compared to ErPC3 treatment alone. The discrepancies between the results from the WST-1 test and flow cytometry may be due to the high standard deviations in the WST-1 test that would preclude the detection of a small combination effect. On the other hand, in cell culture apoptotic cells remain viable at the early stages and die from late apoptosis/necrosis. Thus, early apoptotic cells may be detected as viable in the Wst-1 test, thereby leading to an underestimation of an apoptosis-based cytotoxic drug effect.

In LNCaP cells, the major part of the combination effects seemed to be based on the radiation effects at least when non-toxic concentrations of ErPC3 were used. However, when combining a cytotoxic ErPC3 concentration (50µM) and ionizing radiation, a more prominent reduction in the number of viable cells was achieved compared to either treatment alone. These results were corroborated by the apoptosis determinations: Although LNCaP cells were resistant to apoptosis induction by single treatment with ionizing radiation or low concentrations of ErPC3, a pronounced increase of apoptotic cell death was already observed when combining 12.5 µM ErPC3 and ionizing radiation. The radiation-induced down-regulation of Bcl-2 together with the ErPC3-induced down-regulation of Mcl-1 and p-Akt may be sufficient to overcome the cellular death threshold and to induce apoptotic death of LNCaP cells [16,36]. In PC3-cells, ionizing radiation also decreased cellular Bcl-2 levels but ErPC3 did not reduce the levels of anti-apoptotic Mcl-1. The rather low levels of Bcl-2 in the PC3 cells may explain why the radiation-induced down-modulation of Bcl-2 was of minor importance for the response of PC3-cells to radiotherapy and the combined treatment.

Our novel data emphasize a potential therapeutic benefit of the alkylphosphocholine ErPC3 when used as single drug or in combination with ionizing radiation in prostate cancer. Recent phase-I trials already demonstrated feasibility and tolerability of an intravenous therapy with ErPC3 for patients with advanced human malignancies (personal communication of L. Lindner, Department of Internal Medicine III, Universität München-Großhadern, Germany). Also, the ErPC3-related compound perifosine was well tolerated in clinical trials and displayed clinical activity in hematological malignancies and in a subgroup of patients with recurrent androgen-sensitive prostate cancer [11,30,37]. Moreover, in a recent phase-II-study a single treatment with oral perifosine prolonged the progression free survival and induced a minimal response in a group of patients with Waldenstrom's Macroglobulinema [37]. On the basis of its potential efficacy in patients with recurrent androgen-sensitive tumors, perifosine is currently being developed as an oral Akt inhibitor for prostate cancer [30]. It is expected that a combination therapy with other anti-neoplastic agents or ionizing radiation will further enhance these effects. The clinical use of this class of neoplastic agents is of particular interest because, in contrast to standard genotoxic therapies and ionizing radiation, these drugs target cellular membranes without a direct interaction with the cellular DNA. Consequently, these lipophilic drugs lack bone-marrow toxicity and even exert growth stimulatory effects on hematopoietic progenitor cells [38,39]. The lack of hematotoxicity, and the improved solubility compared to perifosine make ErPC3 the first intravenously applicable alkylphosphocholine for the use in clinical trials allowing a faster drug accumulation in the tumor tissue [40].

In summary, our data underline the relevance of Akt as a therapeutic target in prostate cancer. However, it has to be taken into account that Akt inhibitors with a differential mechanism of action will have differential effects in prostate tumors with a distinct genetic background. A detailed molecular profiling of the tumor cells of each patient as well as the definition of biomarkers which predict the drug response will be of utmost importance to choose the best drug for each patient.

## Abbreviations

ErPC3: erucylphosphohomocholine (erucyl-N: N: N-trimethylpropanolaminphosphate); PARP: Poly-(ADP-ribose)-Polymerase; GSK-3β: glycogen synthase kinase-3β; FKHRL1: forkhead transcription factor like 1; PI3K: phosphatidylinositol-3-kinase; PTEN: phosphatase and tensin homolog on chromosome 10

## Competing interests

The authors declare that they have no competing interests.

## Authors' contributions

JR contributed significantly to data acquisition, participated in the design of the study, data analysis and interpretation, drafting and revising the manuscript. CER contributed significantly to data acquisition and analysis. RH contributed to initial work on ErPC3. HJE provided ErPC3 for all experiments. CB contributed to the design of the study. VJ performed conception and design of the study and substantially contributed to interpretation of data, drafting and critical revision of the manuscript and final approval. All authors read and approved the final manuscript.
